# Regulation of the Activity of the Dual-Function DnaA Protein in *Caulobacter crescentus*

**DOI:** 10.1371/journal.pone.0026028

**Published:** 2011-10-14

**Authors:** Carmen Fernandez-Fernandez, Diego Gonzalez, Justine Collier

**Affiliations:** Department of Fundamental Microbiology, Faculty of Biology and Medicine, University of Lausanne, Lausanne, Switzerland; University of Massachusetts Medical School, United States of America

## Abstract

DnaA is a conserved essential bacterial protein that acts as the initiator of chromosomal replication as well as a master transcriptional regulator in *Caulobacter crescentus*. Thus, the intracellular levels of active DnaA need to be tightly regulated during the cell cycle. Our previous work suggested that DnaA may be regulated at the level of its activity by the replisome-associated protein HdaA. Here, we describe the construction of a mutant DnaA protein [DnaA(R357A)]. The R357 residue in the AAA+ domain of the *C. crescentus* DnaA protein is equivalent to the R334 residue of the *E. coli* DnaA protein, which is required for the Regulatory Inactivation of DnaA (RIDA). We found that the expression of the DnaA(R357A) mutant protein in *C. crescentus*, but not the expression of the wild-type DnaA protein at similar levels, causes a severe phenotype of over-initiation of chromosomal replication and that it blocks cell division. Thus, the mutant DnaA(R357A) protein is hyper-active to promote the initiation of DNA replication, compared to the wild-type DnaA protein. DnaA(R357A) could not replace DnaA *in vivo*, indicating that the switch in DnaA activity once chromosomal replication has started may be an essential process in *C. crescentus.* We propose that the inactivation of DnaA is the main mechanism ensuring that chromosomal replication starts only once per cell cycle. We further observed that the R357A substitution in DnaA does not promote the activity of DnaA as a direct transcriptional activator of four important genes, encoding HdaA, the GcrA master cell cycle regulator, the FtsZ cell division protein and the MipZ spatial regulator of cell division. Thus, the AAA+ domain of DnaA may play a role in temporally regulating the bifunctionality of DnaA by reallocating DnaA molecules from initiating DNA replication to transcribing genes within the unique DnaA regulon of *C. crescentus*.

## Introduction

Faithful chromosomal replication requires regulatory networks that ensure the precise coordination of DNA replication with other cell cycle events. The prokaryotic DnaA protein or the eukaryotic origin replication complex (ORC) control the onset of DNA replication. DnaA and several ORC proteins are ATP-binding proteins that contain AAA+ (ATPase associated with diverse cellular activities) domains of very similar structures, suggesting mechanistic conservation throughout evolution [1], [2]. Binding of ATP to these proteins is thought to induce conformational changes that activate these proteins to promote the initiation of DNA replication at the correct time of the cell cycle. Besides its function as an initiator of bacterial chromosome replication, DnaA is also a transcription factor that binds to many promoters to regulate their activities in diverse bacterial species [3], [4]. It is however still unclear whether oscillations in the levels of DnaA-ATP influence the timing of gene transcription in most cases [5], [6].

In the *Escherichia coli* prokaryotic model, DnaA proteins bind to DnaA boxes located in the origin of replication, but only the ATP-bound form (DnaA-ATP) can initiate replication [1]. Soon after the initiation of DNA replication, the ATPase activity of the AAA+ domain of DnaA is stimulated by the essential Hda protein, resulting in the hydrolysis of the ATP bound to DnaA and thus in the regulatory inactivation of DnaA (RIDA) [1], [7], [8]. Hda supposedly needs to interact with the β-clamp of the DNA polymerase III loaded onto DNA to promote the inactivation of DnaA [9]. Although there exists several other mechanisms that regulate the timing of the initiation of DNA replication in *E. coli* [1], the RIDA system seems to be one of the most important [10], [11]. Consistent with this model, it was shown that ATPase defective mutants of DnaA, and *hda*-deficient cells, display over-initiation phenotypes in *E. coli* [7], [12], [13]. The use of replisome-associated proteins that act as negative regulators of initiation appears quite conserved in bacteria, as indicated by the discoveries of the YabA protein in *Bacillus subtilis* [14], [15] or the HdaA protein in *Caulobacter crescentus* [16].

The *C. crescentus* bacterium undergoes a unique developmental cycle, dividing asymmetrically at each cell cycle, producing two different daughter cells: the stalked cell in S phase, and the swarmer cell in G1 phase until it differentiates into a stalked cell [17], [18]. The G1-to-S transition is controlled by the CtrA protein, an important regulator of the initiation of chromosome replication in *alphaproteobacteria*. This response regulator binds to several sites in the *C. crescentus* origin of replication (*Cori*) to inhibit the initiation of DNA replication, probably by competing with DnaA binding [19]. It also binds to many promoters to regulate their activities [20], [21]. Levels of the active phosphorylated form of CtrA are tightly controlled by the regulated synthesis, phosphorylation and degradation of the CtrA protein, so that it only accumulates in swarmer and pre-divisional cells [17], [18]. A gradient of active CtrA establishes the asymmetry of the initiation of DNA replication between the two future daughter cells before cell division [22]. Active CtrA is however undetectable for about one third of the cell cycle during the stalked cell stage [23]. During the time when no CtrA is detectable, other factors restrict the initiation of chromosomal replication to only once per cell cycle. We previously identified HdaA as one of these factors, showing that the HdaA protein is necessary to ensure that chromosomal replication does not restart a second time during the same cell cycle [16], indicating that HdaA may stimulate the inactivation of DnaA once DNA replication has initiated, by a mechanism similar to the RIDA system in *E. coli*. To further address this possibility, we describe here the creation of a mutant DnaA protein in *C. crescentus*, with an R357A substitution in its AAA+ domain. We found that the expression of this protein caused severe over-initiation of chromosomal replication and blocked cell division in *C. crescentus*, unlike the expression of the wild-type DnaA, indicating that DnaA(R357A) is hyper-active as an initiator of DNA replication. We also showed that DnaA(R357A) cannot substitute for DnaA, indicating that the inactivation of DnaA by its AAA+ domain may be an essential process in *C. crescentus*, to restrict the initiation of DNA replication to only once per cell cycle. We also showed that, surprisingly, the R357A substitution in DnaA does not promote the activity of DnaA as a direct transcriptional activator of four *C. crescentus* genes, encoding HdaA, the GcrA master cell cycle regulator, and the FtsZ cell division protein and the MipZ spatial regulator of cell division, that are all required for normal cell cycle progression.

## Results

### Mutagenesis of a conserved arginine residue in the AAA+ domain of the *C. crescentus* DnaA protein

**Figure 1:**
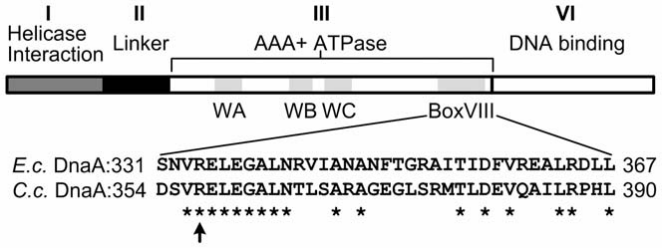
Amino-acid sequence homology between the *C. crescentus* and the *E. coli* DnaA proteins. The upper schematic shows the four typical domains of DnaA proteins [1]. The AAA+ domain contains three conserved Walker motifs indicated as WA, WB, and WC. The bottom schematic shows the homology between the box VIII motif in the AAA+ domains of the *C. crescentus* and *E. coli* DnaA proteins. Amino acid positions are indicated with numbers. R357 from the *C. crescentus* DnaA protein and R334 from the *E. coli* DnaA protein are indicated by a black arrow. *E. c.* indicates *E. coli*, while *C. c.* indicates *C. crescentus*.

Assuming that ATP-DnaA is the active form that initiates chromosomal replication in a bacterial species, DnaA mutants that can bind ATP but that are defective in hydrolysis of the bound ATP may cause over-initiation because the ATP-bound form of DnaA accumulates. In *E. coli*, such a mutant DnaA protein was constructed by a R334A amino-acid substitution [12]. The highly conserved R334 residue lays in the Box VIII sensor 2 motif of the AAA+ domain of DnaA (Fig.1). *In vitro* and *in vivo* experiments demonstrated that this mutant protein can not be inactivated by the RIDA mechanism in *E. coli*, and thus that its expression at moderate levels results in over-initiation of chromosomal replication, unlike the wild-type DnaA protein [12].

**Figure 2:**
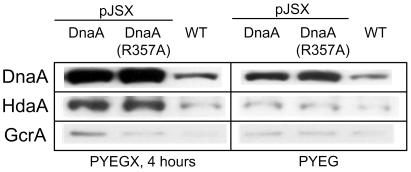
DnaA and DnaA(R357A) are expressed at moderate levels. Strains NA1000 (WT), JC366 (containing pJSX-DnaA) and JC367 [containing pJSX-DnaA(R357A)] were grown to exponential phase in PYE medium plus 0.2% glucose (PYEG) and 0.3% xylose was added (PYEGX) to half of the culture for four hours before cells were collected for immunoblot analysis using antibodies raised against DnaA, HdaA and GcrA.

Our previous discovery of the HdaA protein [16], which is homologous to the *E. coli* Hda protein, suggested that RIDA may also occur in *C. crescentus*, and that this mechanism may restrict the initiation of chromosomal replication to only once per cell cycle in this particular bacterial species. To examine this possibility, we generated a mutant DnaA protein, named DnaA(R357A). The R357A substitution in the AAA+ motif of the *C. crescentus* DnaA protein is equivalent to the previously characterized R334A mutation in the *E. coli* DnaA protein (Fig.1). When expressed from the xylose-inducible *xylX* promoter on a medium copy number plasmid for four hours in rich medium containing xylose, we observed by immunoblot analysis that the levels of DnaA or DnaA(R357A) were approximately four-fold higher than the natural levels of DnaA expressed from its native locus in the wild-type strain (Fig.2). Hence, DnaA(R357A) was expressed at moderate levels that were comparable to the levels of the wild-type DnaA protein expressed from the same vector. We used these conditions to investigate and compare the effects of the expression of DnaA or DnaA(R357A) on cell morphology and chromosome content, and on the expression of four important genes directly activated by DnaA.

### Moderate expression of DnaA(R357A), but not DnaA, blocks cell division

**Figure 3:**
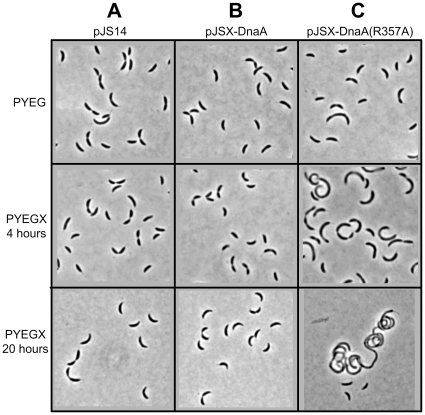
A moderate overproduction of DnaA(R357A), but not DnaA, blocks cell division. Representative phase contrast images from strains JC919 containing the pJS14 empty vector (Panel A), JC366 containing pJSX-DnaA (Panel B) and JC367 containing pJSX-DnaA(R357A) (Panel C) grown to exponential phase in PYE medium plus 0.2% glucose (PYEG) before 0.3% xylose was added (PYEGX) to half of the culture for four hours. Cells maintained in PYEGX for twenty hours are also shown in the bottom panel. All images are at the same scale.

When DnaA was expressed upon xylose induction for four hours or longer time periods from a medium-copy number plasmid in the JC366 strain, we observed by phase contrast microscopy that cells had a quite normal morphology (Fig.3). This observation shows that a four-fold over-expression of DnaA is not preventing cell division. We however still noticed that JC366 cells tended to divide at lower cell mass, giving slightly smaller daughter cells than JC919 cells containing the empty vector.

Next, we analyzed the phenotypes associated with the over-expression of the mutant DnaA(R357A) protein. Phase contrast microscopy four hours after the addition of the xylose inducer showed that JC367 cells expressing DnaA(R357A) were significantly longer than cells over-expressing DnaA and than cells containing the empty control vector (Fig.3). When cells were grown in xylose-containing medium for longer time periods, many cells became very filamentous (Fig.3), indicating a severe reduction in the efficiency of cell division upon expression of DnaA(R357A).

### Expression of DnaA(R357A) leads to over-initiation of chromosomal replication

**Figure 4:**
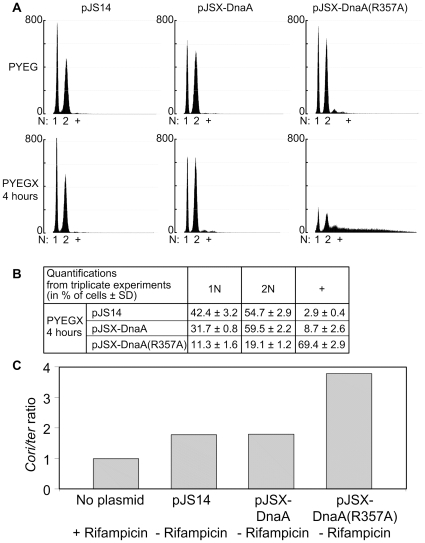
A moderate overproduction of DnaA(R357A), but not DnaA, leads to severe over-initiation of chromosomal replication. (**A**) Cells overproducing DnaA(R357A) accumulate extra chromosomal copies after rifampicin treatment. Representative profiles obtained by flow cytometry analyses of cells from strains JC919 (containing the pJS14 empty vector), JC366 (containing pJSX-DnaA) and JC367 [containing pJSX-DnaA(R357A)] grown to exponential phase in PYE medium plus 0.2% glucose (PYEG) before 0.3% xylose was added (PYEGX) to half of the culture for four hours. Cells were treated with rifampicin for three hours prior to fixing and staining with Vybrant DyeCycle orange. The horizontal axis indicates the number N of complete chromosomes: 1N, 2N or more than 2N (+). The vertical axis indicates the number of cells. (**B**) Quantification of the results of flow cytometry experiments as shown in A. The percentage represents the average proportion of cells containing the indicated number of chromosomes per cell. Results are the averages of data from three independent experiments. The standard deviations are also indicated (SD). (**C**) The graph shows the relative *Cori*/*ter* ratio obtained from Q-PCR on chromosomal DNA extracted from strains JC919 (containing the pJS14 empty vector), JC366 (containing pJSX-DnaA) and JC367 [containing pJSX-DnaA(R357A)]. Cells were grown to exponential phase in PYE medium plus 0.2% glucose before 0.3% xylose was added for four hours. Values were normalized to a control strain, which corresponds to the wild-type strain (NA1000) grown in PYE medium and treated with rifampicin for three hours. Results are the average of data from four independent experiments and standard deviations were always lower than 0.5%.

We previously showed that cells slightly over-expressing DnaA from an integrated second copy of the *dnaA* gene under the control of the *xylX* promoter do not over-initiate DNA replication [16]. We confirmed this result when DnaA was expressed at higher levels from the same promoter on our medium-copy number plasmid (Fig.2). We performed flow-cytometry experiments using a fluorescent dye that labels the DNA, to observe the number of chromosomes in single cells after a rifampicin treatment. We found that only (8.7+/-2.6)% of the cells contained more than two chromosomes, which is just three-fold higher than the control strain containing the empty vector [(2.9+/-0.4)%] (Fig.4A&B). Cells not treated with rifampicin prior to the flow cytometry analysis also had a profile quite comparable to that observed for the control strain (Fig.S1). Measurements of the ratio of *Cori* to *ter* (the replication terminus region) by quantitative-polymerase chain reaction (Q-PCR) from genomic DNA extracted from a population of these cells also confirmed that these cells do not over-initiate chromosomal replication (Fig.4C). Collectively, our data demonstrates that an over-expression of DnaA from a medium-copy number plasmid is not significantly affecting the precise once-per-cell-cycle control of the initiation of chromosome replication in *C. crescentus*. We nevertheless noticed that cells over-expressing DnaA more often accumulated two complete copies of the chromosome after rifampicin treatment, compared to the control cells (Fig.4A&B). This observation may indicate that chromosomal replication starts a little earlier in cells over-expressing DnaA than in control cells.

To determine whether the over-expression of DnaA(R357A) affects the initiation of chromosomal replication, we also performed flow cytometry experiments with JC367 cells. In accordance with our predictions, we observed that (69.4+/-2.9)% of the cells expressing DnaA(R357A) for four hours contained more than two chromosomes after rifampicin treatment (Fig. 4A&B). In fact, many cells even contained numerous incomplete chromosomes per cells after rifampicin treatment. When we performed flow cytometry experiments without a rifampicin treatment, we observed a profile drastically shifted towards a higher amount of DNA content per cell, compared to what we observed with the control strain or with the strain that over-expresses the wild-type DnaA protein at similar levels (Fig.S1). To confirm that these cells over-initiated DNA replication, we also measured the *Cori*/*ter* ratio by Q-PCR from genomic DNA extracted from these cells. Consistent with our single cell flow cytometry results, we observed that the *Cori*/*ter* ratio in cells expressing DnaA(R357A) is about two fold higher than that of cells expressing DnaA as similar levels (Fig.4C). All together, these results indicate that DnaA(R357A) can initiate new rounds of chromosomal replication, but in an uncontrolled manner, suggesting that DnaA(R357A) is hyper-active for its function as an initiator of chromosomal replication. It is very likely that DnaA(R357A) remains bound to ATP, and thus active for replication initiation, at all times of the cell cycle of *C. crescentus*, as it is the case for the DnaA(R334A) protein in *E coli*.

### DnaA(R357A) cannot replace DnaA(WT) *in vivo*

We previously demonstrated that the HdaA protein is essential for normal cell cycle progression in *C. crescentus* and that a Δ*hdaA* strain can not be constructed in the absence of an extra copy of the *hdaA* gene expressed *in trans* [16]. These results suggested that the switch of DnaA activity promoted by HdaA after chromosome replication has initiated could be essential for the viability of *C. crescentus*. If this prediction is correct, it should be very difficult, or impossible, to construct a strain carrying the *dnaA(R357A)* allele as the sole copy of *dnaA* on the *C. crescentus* chromosome. We attempted to construct such a strain by two different and complementary approaches.

First, we tried to construct a [Δ*dnaA*::Ω P*xylX*::*dnaA*(R357A)] strain by generalized transduction using bacteriophage ΦCR30. Two phage lysates were prepared from strain GM2471 containing the Δ*dnaA*::Ω mutation and strain JC125 containing a Δ*CC1613*::Ω mutation. The JC125 strain has no apparent phenotype and grows like the wild-type strain: it was used as a control for transduction efficiency. The Ω cassette confers resistance to spectinomycin and streptomycin. Both phage lysates were used for transduction assays using the NA1000 wild-type strain and the JC323 and JC324 strains containing a copy of the *dnaA* or the *dnaA*(R357A) gene, respectively, under the control of the *xylX* promoter at the *xylX* locus. Upon selection on spectinomycin and streptomycin containing plates, we isolated numerous Δ*CC1613*::Ω transductants in the three different genetic backgrounds, regardless of the presence of the xylose inducer (Table 1), demonstrating that transduction efficiency is not significantly affected by the expression of *dnaA* or *dnaA*(R357A). We also isolated numerous Δ*dnaA*::Ω mutant colonies from our transduction assay into JC323, but only on xylose-containing plates (Table 1). This result demonstrated that the wild-type allele of *dnaA* expressed from the *xylX* promoter is sufficient to complement the unviable Δ*dnaA*::Ω mutation. In contrast, no Δ*dnaA*::Ω mutant colonies were isolated from the transduction assays into the wild-type strain (Table 1), confirming that *dnaA* is essential for viability in *C. crescentus* [24]. Similarly, we were not able to isolate Δ*dnaA*::Ω mutant colonies from the transduction assay into the JC324 strain expressing DnaA(R357A) in the presence of the xylose inducer on PYE plates (Table 1). This result suggested that DnaA(R357A) can not replace DnaA *in vivo*.

**Table 1:** DnaA(R357A) cannot complement for the loss of DnaA.

	Δ*dnaA*::Ω transductants		Δ*CC1613*::Ω transductants	
	PYEG	PYEX	PYEG	PYEX
**NA1000**	-	-	+	+
**NA1000 P** ***xylX*** **::** ***dnaA***	-	+	+	+
**NA1000 P** ***xylX*** **::** ***dnaA*** **(R357A)**	-	-	+	+

We used bacteriophage ΦCR30 to transduce the Δ*dnaA*::Ω or the Δ*CC1613*::Ω alleles into strains NA1000, JC323 and JC324 on PYE medium plus 0.2% glucose (PXEG) or on PYE medium plus 0.3% xylose (PYEX). The + symbol indicates that minimum 400 transductant colonies were isolated. The – symbol indicates that no transductant colonies were isolated.

Second, we tried to exchange the native *dnaA* allele for the *dnaA(R357A)* allele by double recombination using the suicide plasmid pNPTS138-DnaA(R357A) carrying the *dnaA(R357A)* allele. We systematically recovered the *dnaA* allele on the chromosome after re-excision of the plasmid (data not shown), indicating again that the *dnaA(R357A)* allele is probably not viable as the sole copy of *dnaA* on the *C. crescentus* chromosome.

Given that the DnaA(R357A) protein is probably functional to initiate chromosomal replication as indicated by our previous findings (Fig.4), these last genetic evidences suggest that the switch in DnaA activity mediated by its AAA+ domain is an essential process in *C. crescentus* and may also explain why the HdaA protein is essential for normal cell cycle progression in *C. crescentus*.

### DnaA(R357A) is not more active than DnaA to promote the transcription of four important *C. crescentus* genes

DnaA is not only the initiator of chromosomal replication in nearly all bacteria, but it also acts as an important transcriptional regulator by directly binding to promoters in multiple bacterial species. In *B. subtilis*, for example, it was estimated that DnaA directly regulates the transcription of about 50 different genes [4], while DnaA directly regulates the transcription of minimum 13 genes in *C. crescentus* [3], [16], [25]. One of the still outstanding questions regarding DnaA activity as a transcription factor is whether the nucleotide bound to DnaA often influences its binding and activity at DnaA-regulated promoters in diverse bacterial species [2].

We investigated whether the DnaA(R357A) protein may also be hyper-active to promote transcription from four well-characterized DnaA-activated promoters in *C. crescentus*. These are: (i) the *hdaA* promoter, controlling the expression of the HdaA repressor of the initiation of chromosomal replication [16]; (ii) the *gcrA* promoter, controlling the expression of the GcrA master regulator of the *C. crescentus* cell cycle [3], [25], [26]; (iii) the *ftsZ* promoter, controlling the expression of the FtsZ cell division protein [3], [27], [28]; (iv) the *mipZ* promoter, controlling the expression of the MipZ spatial regulator of cell division [3], [29]. We chose these four promoters because they have a quite different structure with respect to the position and the number of DnaA boxes that they contain. Indeed, the *gcrA* and *mipZ* promoters contain only one DnaA box, the *ftsZ* promoter contains two DnaA boxes, and the *hdaA* promoter contains up to six DnaA boxes [16]. In addition, the four genes that we selected are essential or required for normal cell cycle progression in *C. crescentus*.

We first compared by immunoblot analysis the amounts of GcrA and HdaA that accumulated in cell extracts from the wild-type strain and from the strain that over-expresses DnaA. We found that GcrA and HdaA accumulated at higher levels in DnaA over-expressing cells than in wild-type cells, although the effect was more pronounced for HdaA than for GcrA (Fig.2). HdaA accumulated at similar levels in cells expressing DnaA(R357A) compared to cells expressing DnaA (Fig.2). In contrast, GcrA accumulated at lower levels in cells constitutively expressing DnaA(R357A) compared to cells expressing DnaA (Fig.2). Altogether, these observations suggested that DnaA(R357A) is not more active than DnaA to promote the transcription of at least certain genes that belong to the direct DnaA regulon.

**Figure 5:**
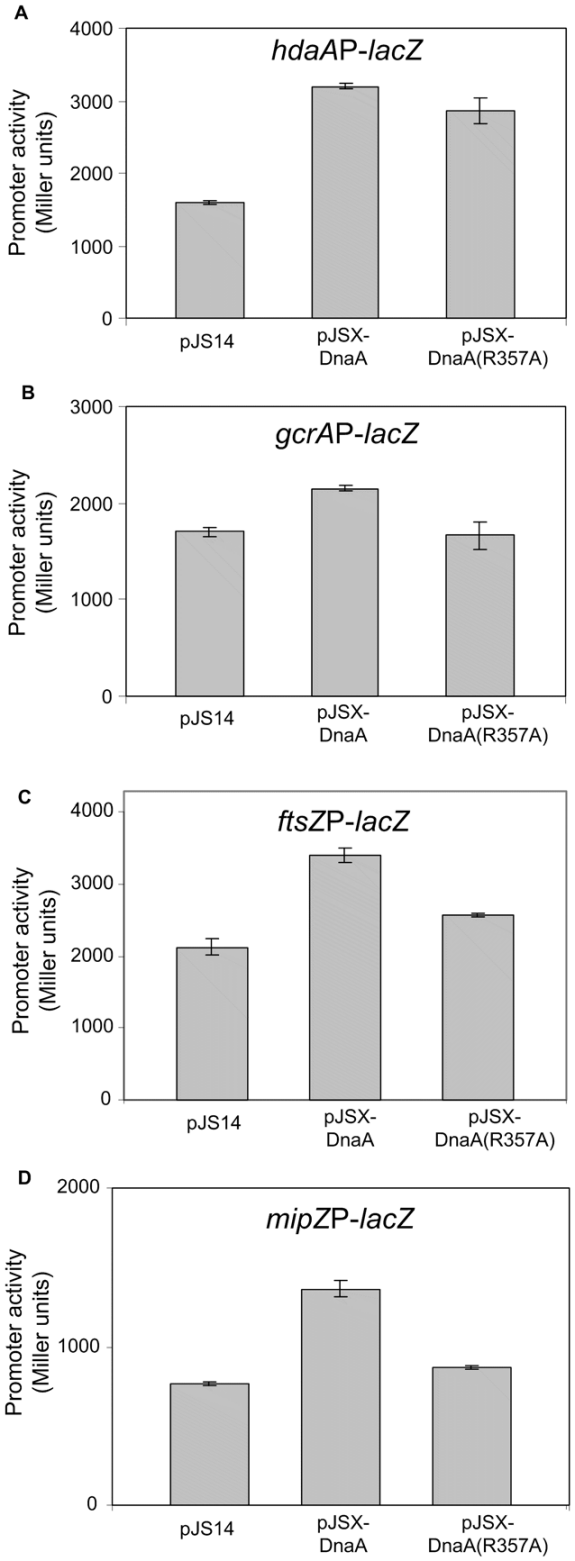
Effect of DnaA and DnaA(R357A) on the transcription of four DnaA-regulated genes. **(A) Effect on the transcription of** ***hdaA,*** **encoding the inhibitor of DnaA.** The graph shows the relative β-galactosidase activities from *placZ290-hdaA*P(WT) in strains JC964 (containing pJS14), JC973 (containing pJSX-DnaA) and JC974 [containing pJSX-DnaA(R357A)]. **(B) Effect on the transcription of** ***gcrA*** **, encoding a master transcriptional regulator.** The graph shows the relative β-galactosidase activities from *placZ290-gcrA*P in strains JC971 (containing pJS14), JC972 (containing pJSX-DnaA) and JC975 [containing pJSX-DnaA(R357A)]. **(C) Effect on the transcription of** ***ftsZ,*** **encoding the cell division protein FtsZ.** The graph shows the relative β-galactosidase activities from *placZ290-ftsZ*P in strains JC1006 (containing pJS14), JC1007 (containing pJSX-DnaA) and JC1008 [containing pJSX-DnaA(R357A)]. **(D) Effect on the transcription of** ***mipZ,*** **encoding a spatial regulator of FtsZ assembly.** The graph shows the relative β-galactosidase activities from *placZ290-ftsZ*P in strains JC1037 (containing pJS14), JC1038 (containing pJSX-DnaA) and JC1039 [containing pJSX-DnaA(R357A)]. In each case, cells were grown to exponential phase in PYE medium plus 0.2% glucose, before 0.3% xylose was added for 4 hours. Error bars indicate the standard deviations.

To confirm that the activity of DnaA as a transcriptional regulator of our four selected genes is not increased by the R357A mutation, we introduced a low copy number plasmid carrying a transcriptional fusion between the *hdaA* promoter, the *gcrA* promoter, the *ftsZ* promoter or the *mipZ* promoter and the *lacZ* gene [16], [25], [26], into the strains that over-express DnaA or DnaA(R357A). We measured β-galactosidase activities as an indication of promoter activities (Fig.5). Four hours after xylose addition into the medium, we observed that β-galactosidase activities from each promoter that we tested were higher in cells over-expressing DnaA than in wild-type cells. Consistent with previous data [16], [25], these results confirm that DnaA activates the transcription of all four genes. Next, we compared the effect of the over-expression of DnaA or DnaA(R357A) on the activities of these four promoters. Surprisingly, we observed that the activation of the transcription from these four promoters was never higher when DnaA(R357A) rather than DnaA was over-expressed (Fig.5). These interesting results indicate that DnaA(R357A) is not more efficient than DnaA to activate the transcription of these four important genes. Thus, the R357A mutation in DnaA uncouples the ability of DnaA to initiate DNA replication from its activity as a transcription factor regulating minimum four genes, only leading to increased activity in the initiation of DNA replication. In addition, we observed that DnaA(R357A) is even less efficient than DnaA to activate the transcription of *gcrA*, *ftsZ* and *mipZ* (Fig.5B&C&D), suggesting that the switch in the activity of DnaA that takes place at the time when DNA replication is initiated, may promote the expression of these genes.

## Discussion

In this study, we showed that the DnaA(R357A) mutant protein in *C. crescentus* retains its ability to promote the initiation of chromosomal replication *in vivo*, and is even hyper-active as an initiator compared to the wild-type DnaA protein, as indicated by the severe over-replication phenotype of cells that over-express DnaA(R357A) (Fig.4). In addition, we showed that the DnaA(R357A) protein cannot replace DnaA (Table 1), suggesting that the inactivation of the initiator DnaA is an essential process in *C. crescentus*. In contrast, we observed that the activity of DnaA(R357A) as a transcription factor that stimulates the transcription of four genes is not higher than that of DnaA (Fig.5), indicating that the AAA+ domain of DnaA may not inactivate DnaA as a transcriptional regulator of these genes in *C. crescentus*. Below, we discuss the role of the AAA+ domain of DnaA in the regulation of both activities of DnaA in the control of the *C. crescentus* cell cycle.

### Model for a RIDA system in *C. crescentus*

**Figure 6:**
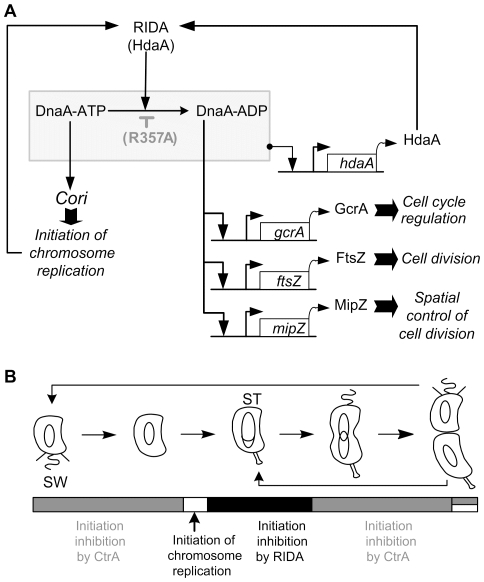
Control of the initiation of chromosomal replication in *C. crescentus*. **(A) Model for a RIDA system in** ***C. crescentus*** **.** A schematic connecting the regulation of the activity of DnaA with processes regulated by DnaA is shown. We propose that DnaA associates with ATP soon after its synthesis and thus promotes the initiation of chromosomal replication in the absence of active CtrA. Once replication is initiated, the ATPase activity of DnaA is stimulated by HdaA assembled onto the replisome, to hydrolyse the ATP bound to DnaA into ADP, resulting in the inactivation of DnaA as an initiator of DNA replication (RIDA). The *hdaA*, *gcrA, ftsZ* and *mipZ* promoters are all directly activated by DnaA. DnaA-ADP may be more efficient than DnaA-ATP when activating the *gcrA*, *ftsZ* and *mipZ* promoters. The effect of the R357A mutation in DnaA is also shown on the schematic (accumulation of DnaA-ATP). **(B) Model for the temporal control of the initiation during the** ***C. crescentus*** **cell cycle.** A schematic of the *C. crescentus* cell cycle is shown. Theta structures inside the cells indicate replicating DNA. SW indicates swarmer cells. ST indicates stalked cells. In swarmer cells, active CtrA is abundant and it blocks the initiation of DNA replication. Once CtrA is degraded during the swarmer-to-stalked cell transition, active DnaA initiates DNA replication. Immediately after, DnaA is inactivated by a RIDA process involving HdaA and the replisome, thus preventing the start of new rounds of replication in stalked cells. In predivisional cells, active CtrA re-accumulates, to block the initiation of DNA replication in the future swarmer progeny. Overall, CtrA determines when and where the initiation of DNA replication can start, while the RIDA system ensures that it starts only once per cell cycle.

The R357A substitution in the AAA+ motif of the *C. crescentus* DnaA protein is equivalent to the previously characterized R334A mutation in the *E. coli* DnaA protein that inhibits RIDA and the intrinsic ATPase activity of DnaA *in vivo* and *in vitro* [12], [30]. It is thus likely that the R357 residue in the AAA+ domain of the *C. crescentus* DnaA protein participates in the hydrolysis of an ATP bound to DnaA, to inactivate DnaA immediately following the initiation of chromosome replication (Fig.6). Consistent with this model, the *C. crescentus* DnaA(R357A) protein would be bound to ATP at all times of the cell cycle, as it is the case for the *E. coli* DnaA(R334A) protein. Then, DnaA-ATP would initiate chromosome replication whenever and wherever active CtrA is absent, leading to *C. crescentus* cells that have undergone additional chromosome replication initiations like we observed (Fig.4). The *C. crescentus* HdaA protein may be a functional homolog of the *E. coli* Hda protein, stimulating the ATPase activity of the AAA+ domain of DnaA when bound to the replisome. In agreement with this model, we observed that the phenotype of HdaA-depleted cells [16] resembles that of DnaA(R357A) over-expressing cells (Fig. 3 and 4), and that the HdaA protein dynamically co-localizes with the replisome [16]. Our results suggest that the inactivation of the initiator DnaA by the hydrolysis of the ATP bound to DnaA by its AAA+ domain is an essential process in *C. crescentus*, as we were not able to replace the wild-type *dnaA* allele by the mutant *dnaA(R357A)* allele on the *C. crescentus* chromosome (Table 1 and data not shown). This could then explain why the HdaA protein is also essential for normal cell cycle progression in *C. crescentus* [16]. When DnaA(R357A) was expressed together with DnaA in strains such as JC367 or JC324, DnaA was probably competing with DnaA(R357A) when binding the *Cori* prior to the initiation of chromosomal replication, thereby maintaining cells alive by the inactivation of the wild-type subset of the multiple DnaA molecules bound to the *Cori* after the initiation of chromosomal replication.

The intracellular levels of DnaA fluctuate during the *C. crescentus* cell cycle, being the most abundant at the time when chromosomal replication is initiated during the swarmer-to-stalked cell transition [25], [31]. Both the transcription of the *dnaA* gene and the proteolysis of the DnaA protein are temporally regulated, to ensure that DnaA accumulates the most at the right time of the cell cycle [31], [32], [33], [34]. Our data suggest that the proteolysis of DnaA in stalked cells is not sufficient to inhibit the initiation of chromosomal replication after the first round of replication has started, and that the activity of DnaA also needs to be down-regulated at that moment of the cell cycle for normal cell cycle progression. Consistent with this proposal, we found that the constitutive expression of a DnaA(R357A) mutant protein resulted in severe over-initiations of chromosomal replication (Fig.4), while the constitutive expression of DnaA did not. Jonas *et al.* recently reported that the over-production of an M2-tagged DnaA protein resulted in over-initiation of chromosomal replication in *C. crescentus* [35], unlike what we observed when we over-produced the native DnaA protein. This difference may be attributable to the artificial addition of the M2 epitope to the DnaA protein by Jonas *et al*., or to differences in growth conditions that may promote DnaA accumulation at higher levels.

### CtrA and the RIDA system have complementary functions in the control of the initiation of chromosomal replication in *C. crescentus*

The CtrA master regulator is conserved in alpha-proteobacteria but is not found in entero-bacteria like *E. coli*. In *C. crescentus*, it acts as an inhibitor of the initiation of chromosomal replication that is present and active in swarmer cells and in the swarmer compartment of pre-divisional cells (Fig.6B). Thus, CtrA essentially inhibits replication by binding to the origin of non-replicating chromosomes [19], [22], [36]. Accordingly, we observed that (11.3+/-1.6)% of the cells expressing DnaA(R357A) still contained only one chromosome by flow cytometry analysis after rifampicin treatment (Fig.3). This observation suggests that CtrA is still able to inhibit the initiation of DNA replication in the presence of the hyper-active DnaA(R357A) mutant protein in swarmer cells. HdaA protein co-localizes with the replisome assembled at the replication forks once DNA replication is ongoing [16]. In *E. coli*, Hda needs to interact with the active replisome to stimulate the AAA+ domain of DnaA and thereby promote the inactivation of DnaA [9]. By analogy, we propose that the *C. crescentus* RIDA system inactivates DnaA as soon as chromosomal replication has started, and maybe until the end of the replication process (Fig.6B). This coincides with the period of the cell cycle when active CtrA is poorly abundant or absent, suggesting that RIDA and the activity of CtrA have complementary roles in controlling the timing of the initiation of chromosomal replication. We propose that active CtrA determines when the initiation of chromosomal replication can start, while the RIDA system ensures that chromosomal replication starts only once per cell cycle in *C. crescentus*.

### Is the RIDA system involved in the transcriptional control of DnaA-regulated genes in *C. crescentus*?

Besides its function as the initiator of chromosomal replication, DnaA is also a master transcriptional regulator in *C. crescentus* [3]. DnaA activates the transcription of about forty different genes at the swarmer-to-stalked cell transition, by directly binding to minimum thirteen different promoters. Genes whose expression is directly regulated by DnaA encode proteins involved in multiple processes essential for cell cycle progression. Examples are the FtsZ protein required for cell division, the NrdB protein involved in nucleotide synthesis, and the cell polarity factor PodJ.

To explore the potential role of the AAA+ domain of DnaA in the regulation of the activity of DnaA as a transcription factor, we compared the effect of the over-expression of DnaA or DnaA(R357A) on the activities of several promoters driving the expression of proteins involved in the RIDA system, in cell cycle regulation and in cell division in *C. crescentus*. Interestingly, the selected genes are not regulated by DnaA in *E. coli*. We found that DnaA(R357A) is not more active than DnaA to activate the transcription of each selected genes (Fig.5), suggesting that DnaA-ATP is not more active than DnaA-ADP when acting as a transcription factor regulating the expression of these genes (Fig.6). Thus, the DnaA mutant we generated decouples the activity of DnaA as a transcription factor regulating several genes from its activity as an initiator of DNA replication.

We also observed that DnaA(R357A) was less efficient than DnaA in activating *gcrA*, *ftsZ* and *gcrA* transcription (Fig.5B&C&D). This effect is particularly striking for the *gcrA* and *mipZ* promoters, which are not significantly activated by the expression of DnaA(R357A), compared to the control strain containing an empty vector (Fig.5B). One possible interpretation is that these three promoters may be more efficiently activated by DnaA-ADP than by DnaA-ATP (Fig.6). Genes specifically activated by DnaA-ADP may then wait for the initiation of DNA replication to be activated by DnaA, thereby coordinating the initiation of DNA replication with the expression of specific genes. Consistently, the transcription of *gcrA* is at its maximum in stalked cells, right after the initiation of DNA replication [25], [26]. Also, GcrA activates the transcription of genes whose products are involved in the elongation of DNA replication and in chromosome segregation [26]. Thus, it may be logical for these genetic modules to be expressed only after the initiation of DNA replication. We propose that the AAA+ domain of DnaA plays a role in temporally regulating the bifunctionality of DnaA by reallocating DnaA molecules from initiating DNA replication to transcribing genes within the DnaA regulon.

The extent to which the regulation of the activity of DnaA may affect the regulation of other genes directly controlled by DnaA remains unkown in *C. crescentus* and future detailed investigations studying the regulation of each gene will be required to determine in which cases the AAA+ domain of DnaA may regulate the activity of DnaA as a transcription factor. In other bacterial species, there are now several examples of DnaA-regulated genes whose expression was shown to depend on both the concentration and the nucleotide-bound state of DnaA [2], [5], [37], [38], but the role of the nucleotide bound to DnaA in the regulation of the activity of DnaA as a transcription factor still remains poorly understood in most cases [2]. Thus, the full extend to which DnaA is utilized to regulate the timing of gene expression during the bacterial cell cycle is an interesting avenue for future research and *C. crescentus* is an ideal model to study such questions.

## Materials and Methods

### Bacterial strains and growth conditions

*C. crescentus* strains were grown in PYE complex media [39] at 28°C. Plasmids and strains used are listed in Table 2 and Table 3. Antibiotics used for *C. crescentus* liquid cultures include rifampicin (15 µg/mL), chloramphenicol (1 µg/mL), kanamycin (5 µg/mL) and oxytetracycline (1 µg/mL). Antibiotics used for *E. coli* liquid cultures include chloramphenicol (20 µg/mL), kanamycin (30 µg/mL) and oxytetracycline (12 µg/mL). Plasmids were mobilized from *E. coli* S17-1 [40] into *C. crescentus* by bacterial conjugation or introduced by electro-transformation. Bacteriophage ΦCR30 was used for general transduction into *C. crescentus.*

**Table 2:** Strains.

Strains	Description	Reference/source
***Escherichia coli***		
TOP10	Cloning strain	Invitrogen
S17	RP4, Tc::Mu Km::Tn7	[40]
***Caulobacter crescentus***		
NA1000	Synchronizable derivative of wild-type strain	[45]
GM2471	NA1000 Δ*dnaA*::Ω P*xylX*::*dnaA*	[24]
JC125	NA1000 Δ*CC1613*::Ω	This study
JC919	NA1000 pJS14	This study
JC366	NA1000 pJSX-DnaA	This study
JC367	NA1000 pJSX-DnaA(R357A)	This study
JC323	NA1000 P*xylX*::*dnaA*	This study
JC324	NA1000 P*xylX*::*dnaA*(R357A)	This study
JC964	JC919 p*lacZ*290-*hdaA*P(WT)	This study
JC973	JC366 p*lacZ*290-*hdaA*P(WT)	This study
JC974	JC367 p*lacZ*290-*hdaA*P(WT)	This study
JC971	JC919 p*lacZ*290-*gcrA*P	This study
JC972	JC366 p*lacZ*290-*gcrA*P	This study
JC975	JC367 p*lacZ*290-*gcrA*P	This study
JC1006	JC919 p*lacZ*290-*ftsZ*P	This study
JC1007	JC366 p*lacZ*290-*ftsZ*P	This study
JC1008	JC367 p*lacZ*290-*ftsZ*P	This study
JC1037	JC919 p*lacZ*290-*mipZ*P	This study
JC1038	JC366 p*lacZ*290-*mipZ*P	This study
JC1039	JC367 p*lacZ*290-*mipZ*P	This study

**Table 3:** Plasmids.

Plasmids	Description	Reference/source
pCR-BluntII-TOPO	Cloning plasmid	Invitrogen
pDnaA(R357A)	*dnaA(R357A)* in pCR-BluntII-TOPO	This study
pJS14	Mid-copy number replicating plasmid	J. Skerker, unpublished
pJSX-DnaA	*dnaA(WT)* under the control of the *xylX* promoter in pJS14	[16]
pJSX-DnaA(R357A)	*dnaA(R357A)* under the control of the *xylX* promoter in pJS14	This study
pXGFP4C1	Integrating plasmid	D. Alley, unpublished
pX-DnaA	*dnaA(WT)* under the control of the *xylX* promoter in pXGFP4C1	[16]
pX-DnaA(R357A)	*dnaA(R357A)* under the control of the *xylX* promoter in pXGFP4C1	This study
pNPTS138	Integrating plasmid containing the *sacB* gene	D. Alley, unpublished
pHP45Ω	Vector carrying a Spec^R^/Strep^R^ cassette (Ω)	[46]
pBOR	2-kb EcoRI fragment from pHP45Ω ligated into EcoRI-digested pBluescript	C. Stevens, unpublished
pNPTS138-DnaA(R357A)	*dnaA(R357A)* cloned into pNPTS138	This study
pNPTS138-Δ*CC1613*::Ω	The regions upstream and downstream of the *CC1613* coding sequence flanking an Ω cassette, cloned into pNPTS138	This study
p*lacZ*290	Low-copy number plasmid to create transcriptional fusions with *lacZ*	[47]
p*lacZ*290-*hdaA*P(WT)	*lacZ* gene under the control of the wild-type *hdaA* promoter in p*lacZ*290	[16]
p*lacZ*290-*gcrA*P	*lacZ* gene under the control of the wild-type *gcrA* promoter in p*lacZ*290	[25], [26]
p*lacZ*290-*ftsZ*P	*lacZ* gene under the control of the *ftsZ* promoter in p*lacZ*290	This study
p*lacZ*290-*mipZ*P	*lacZ* gene under the control of the *mipZ* promoter in p*lacZ*290	This study

### Plasmids constructions

#### Construction of pDnaA(R357A)

Oligos 5′-CCCATATGACCATGAAGGGCGGGGTTGCC-3′, 5′-CCGGATCCTTAGCCCCGCAGCTTGCGCGT-3′, 5′-CACCGACAGCGTCGCCGAGCTGGAAGGC-3′ and 5′- GCCTTCCAGCTCGGCGACGCTGTCGGTG-3′ were used to create a mutated *dnaA* coding sequence [dnaA(R357A)] from a two-step PCR amplification procedure using CB15N genomic DNA. The corresponding PCR product was cloned into pCR-BluntII-TOPO (Invitrogen).

#### Construction of the pX-DnaA(R357A) plasmid

pDnaA(R357A) was digested by NdeI and BamHI to extract the mutant *dnaA* insert, which was cloned into NdeI-BamHI-digested pXGFP4C1 (with the *gfp* gene eliminated).

#### Construction of the pJSX-DnaA plasmid

Oligos 5′-AAGGTACCCAGCCGATCAGGCGGAACTGG-3′ and 5′- CCGGATCCTTAGCCCCGCAGCTTGCGCGT-3′ were used to amplify P*xylX*::*dnaA* from pX-DnaA. The PCR product was digested by KpnI and BamHI, and cloned into KpnI-BamHI-digested pJS14.

#### Construction of the pJSX-DnaA(R357A) plasmid

same as construction of pJSX-DnaA, except that pX-DnaA(R357A) was used as template DNA for the PCR amplification.

#### Construction of the pNPTS138-Δ*CC1613*::Ω plasmid

The *CC1613* downstream region was PCR-amplified using primers 5′-CCGGATCCCCGCGAATCTCGCACTGA-3′ and 5′-CCGGGCTAGCGCGGCGATTGGCGAGGTG-3′. The 500 base pair product was digested with *Nhe*I and *Bam*HI, and cloned into a *Nhe*I-*Bam*HI-digested pNPTS138 plasmid, giving pNPTS138-*CC1613*down. The *CC1613* upstream region was PCR-amplified using primers 5′-GGTAAGCTTCGCGCCGCGACAGGCCTGGG-3′ and 5′-CCGGATCCGGCGAGACGAGAATTCATGGCG-3′. The 500 base pair product was then digested with *Hind*III and *Bam*HI, and cloned into *Bam*HI-*Hind*III-digested pNPTS138-*CC1613*down, giving the pNPTS138-Δ*CC1613* plasmid. The 2 kb BamHI fragment from pBOR, containing the Spec^R^/Strep^R^ cassette (Ω), was ligated into BamHI-digested pNPTS138-Δ*CC1613*.

#### Construction of the pNPTS138-*dnaA*(R357A) plasmid

The*dnaA(R357A)* coding sequence was PCR-amplified from pDnaA(R357A) using primers 5′-GGTAAGCTTACCATGAAGGGCGGGGTTGCC-3′ and 5′-ACGCGCAAGCTGCGGGGCTAAGGATCCGG-3′. The product was then digested with BamHI and HindIII, and cloned into *Bam*HI-*Hind*III-digested pNPTS138.

#### Construction of the p*lacZ*290-*ftsZ*P plasmid

The *ftsZ* promoter region was PCR-amplified using primers 5′- GGAATTCCAGCCAGCTGGCCGGTGTGC-3′ and 5′-AACTGCAGGGGACCCTCGCGTCCTTACGC-3′. The product was then digested with EcoRI and PstI, and cloned into EcoRI-PstI-digested p*lacZ*290.

#### Construction of the p*lacZ*290-*mipZ*P plasmid

The *mipZ* promoter region was PCR-amplified using primers 5′-CGGAATTCTCGGGGCCTCCACGCAAACTGG-3′ and 5′-AACTGCAGGGCTCGGATCCTTCTGCGTCGC-3′. The product was then digested with EcoRI and PstI, and cloned into EcoRI-PstI-digested p*lacZ*290.

### Strain constructions

Construction of the JC323 and JC324 strains: Plasmids pX-DnaA and pX-DnaA(R357A) were integrated into the *xylX* promoter [41] of strain NA1000 by single integration events, giving strains JC323 and JC324 respectively.

#### Construction of the JC125 strain

To integrate plasmid pNPTS138-Δ*CC1613*::Ω into the *C. crescentus* chromosome by single homologous recombination, plasmid pNPTS138-Δ*CC1613*::Ω was introduced into strain NA1000 by conjugation, selecting for kanamycin-resistant colonies with the plasmid integrated at the *CC1613* locus by PCR. The resulting strain was grown to stationary phase in PYE medium lacking kanamycin. Cells were plated on PYE + sucrose 3% and incubated at 28°C. Single colonies were picked and transferred in parallel onto plain PYE plates and PYE plates containing kanamycin. Kanamycin-sensitive and Spec^R^/Strep^R^ colonies, which had lost the integrated plasmid due to a second recombination event, were then checked for the presence of the Δ*CC1613*::Ω allele by colony PCR.

### Immunoblot analysis

DnaA, GcrA and HdaA proteins were resolved on 10%, 15% and 12% SDS/PAGE, respectively [42]. Gels were electrotransfered to a PVDF membrane (Millipore). Immunodetection was performed with polyclonal antibodies. Anti-HdaA and anti-GcrA sera were diluted 1∶2000, anti-rabbit-conjugated to horse-radish peroxidase (Sigma Aldrich) and anti-DnaA sera were diluted 1∶10000. A chemiluminescent reagent (PerkinElmer, Wellesley, MA) and Kodak (Rochester, NY) Bio-Max MR films were used. Images were processed with Photoshop (Adobe, Mountain View, CA), and relative band intensities were determined by using ImageJ software version 1.43.

### Flow cytometry analysis

Rifampicin-treated cells were fixed and stained with the DNA-binding Vybrant DyeCycle Orange (Invitrogen), as previously described [43]. Rifampicin treatment of cells blocks the initiation of chromosomal replication, but allows ongoing rounds of replication to finish. Fixed cells were analyzed using a FACS-Calibur (BD Biosciences, Erembodegem, Belgium) cytometer, equipped with an air-cooled argon laser (488 nm). Flow cytometry data were acquired using the CellQuest software. 30000 cells were analyzed from each biological sample. To quantify the results (Fig.4B), the proportion of cells having 1N, 2N or >2N chromosomes was estimated on the basis of the fluorescence area (FL2-A) given by the flow cytometer for each cell. The data were normalized so that the fluorescence value for the maximum of the 1N peak is equal for all data sets. The average difference N between the 2N and the 1N peak maximum was estimated from representative data sets. In each data set, all cells whose fluorescence is greater than 0.5N and smaller than 1.5N fall in the 1N category; all cells whose fluorescence is greater than 1.5N and smaller than 2.5N fall in the 2N category; all cells whose fluorescence is greater that 2.5N fall in the >2N category.

### Q-PCR to measure *Cori/ter* ratios

Cells were grown to exponential phase in PYE medium plus 0.2% glucose, before 0.3% xylose was added for 4 hours. Cells were harvested and chromosomal DNA was extracted using Wizard Genomic DNA Purification Kit (Promega), 2 µl of lysozyme (20 mg/ml) were used instead of Cell Lysis Solution. The following primer sets were used for the Q-PCR reaction: Cori_fwd (5′-CGCGGAACGACCCACAAACT-3′) and Cori_rev (5′-CAGCCGACCGACCAGAGCA-3′) targeting a region close to the origin (*Cori*) ; Ter_fwd (5′-CCGTACGCGACAGGGTGAAATAG-3′) and Ter_rev (5′-GACGCGGCGGGCAACAT-3′), targeting a region close to the terminus (*ter*). Reactions were run using SYBR Green Supermix (KAPA biosystems) and a Mx3005P Instrument (Stratagene) in a volume of 20 µl, containing 10 µl of Supermix, 2 µl of each pair of primers (concentration 4 µM) and 8 µl of DNA. Reactions were run in 4 replicates. Efficiency of each primer set was calculated by performing a standard curve with 4 different dilutions of primers. For quantification of the results, a calibrator-normalized relative analysis was performed using MxPro qPCR Software (Stratagene) for determining the relative abundance of the chromosomal *Cori* and *ter* sites in each of the samples. The results were normalized to the *Cori*/*ter* ratio of the wild-type control strain (NA1000) treated with rifampicin for three hours, whose *Cori*/*ter* ratio is expected to be close to 1.

### Microscopy

Cells were immobilized using a thin layer of media + 1% agarose. Phase contrast microscopy images were taken with a Plan-Apochromat 100X/1.45 oil Ph3 objective on an AxioImager M1 microscope (Zeiss) with a cascade 1K EMCCD camera (Photometrics) controlled through Metamorph 7.5 (Universal Imaging, Downingtown, PA). Images were processed using Adobe Photoshop and Metamorph 7.5.

### Promoter activity assays

The β-galactosidase activity of strains containing p*LacZ*290 derivatives was assayed from exponential phase cultures in PYE media, as previously described [44]. β-galactosidase activities represent the average of minimum three independent assays.

## Supporting Information

Figure S1 **A moderate overproduction of DnaA(R357A), but not DnaA, leads to severe over-initiation of chromosomal replication.** Representative profiles obtained by flow cytometry analyses of cells from strains JC919 (containing the pJS14 empty vector), JC366 (containing pJSX-DnaA) and JC367 [containing pJSX-DnaA(R357A)] grown to exponential phase in PYE medium plus 0.2% glucose (PYEG) before 0.3% xylose was added (PYEGX) to half of the culture for four hours. Cells were fixed and stained with Vybrant DyeCycle orange. The horizontal axis indicates the number N of complete chromosomes: 1N, 2N or more than 2N (+). The vertical axis indicates the number of cells.
(DOC)
