# Supplementary material for: Regulation of the Activity of the Dual-Function DnaA Protein in Caulobacter crescentus
Source: PLoS One. 2011 Oct 14;6(10):e26028. doi: 10.1371/journal.pone.0026028 (PMC3193534; doi:10.1371/journal.pone.0026028)
Supplement: Figure S1 — A moderate overproduction of DnaA(R357A), but not DnaA, leads to severe over-initiation of chromosomal replication. Representative profiles obtained by flow cytometry analyses of cells from strains JC919 (containing the pJS14 empty vector), JC366 (containing pJSX-DnaA) and JC367 [containing pJSX-DnaA(R357A)] grown to exponential phase in PYE medium plus 0.2% glucose (PYEG) before 0.3% xylose was added (PYEGX) to half of the culture for four hours. Cells were fixed and stained with Vybrant DyeCycle orange. The horizontal axis indicates the number N of complete chromosomes: 1N, 2N or more than 2N (+). The vertical axis indicates the number of cells. (DOC) [file pone.0026028.s001.doc]

**
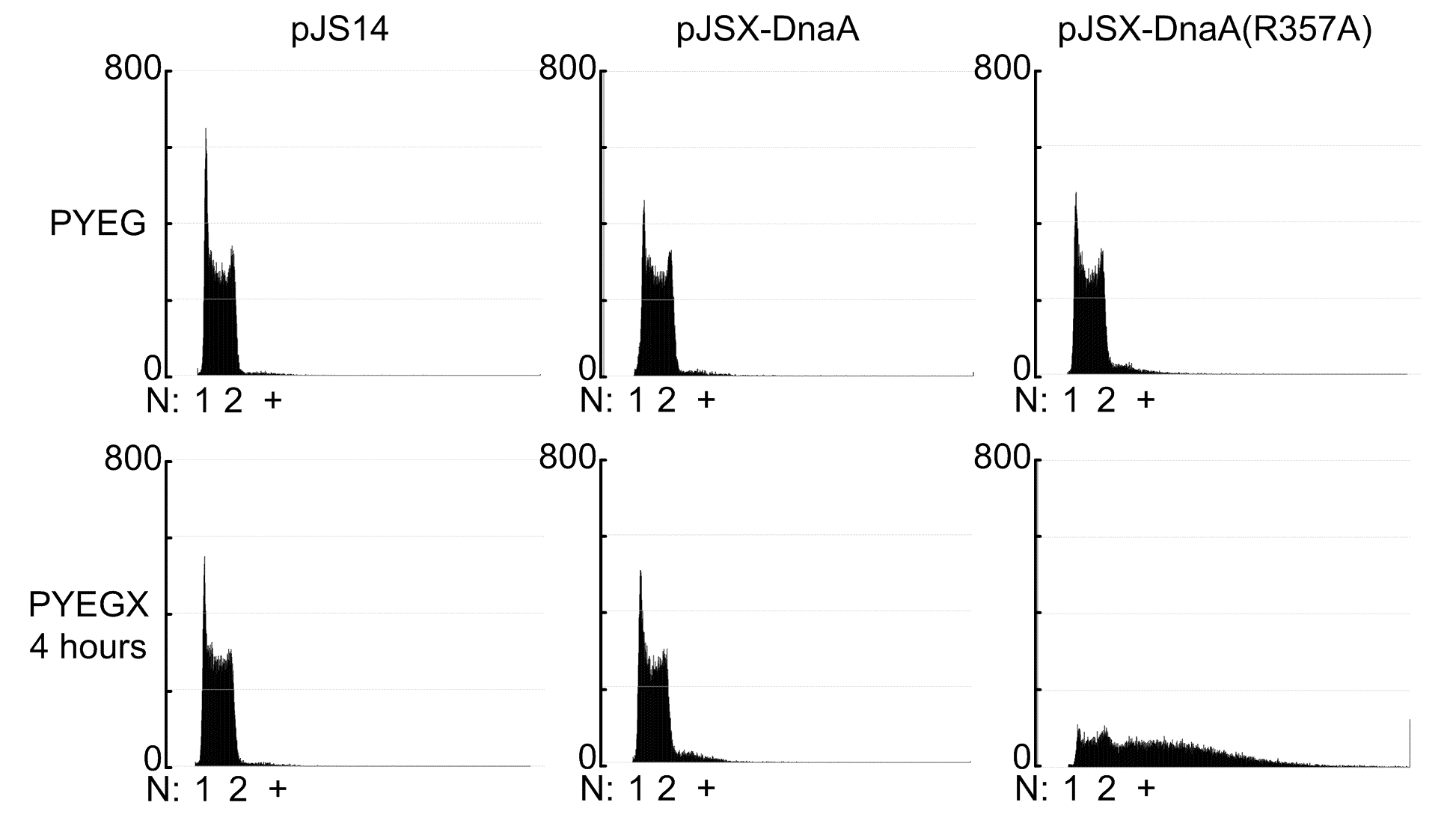
**

**Figure S1: A moderate overproduction of DnaA(R357A), but not DnaA, leads to severe over-initiation of chromosomal replication.** Representative profiles obtained by flow cytometry analyses of cells from strains JC919 (containing the pJS14 empty vector), JC366 (containing pJSX-DnaA) and JC367 [containing pJSX-DnaA(R357A)] grown to exponential phase in PYE medium plus 0.2% glucose (PYEG) before 0.3% xylose was added (PYEGX) to half of the culture for four hours. Cells were fixed and stained with Vybrant DyeCycle orange. The horizontal axis indicates the number N of complete chromosomes: 1N, 2N or more than 2N (+). The vertical axis indicates the number of cells.
